# Knowledge and attitudes of primary care physicians regarding childhood hearing loss in Malaysia

**DOI:** 10.1371/journal.pone.0295972

**Published:** 2023-12-21

**Authors:** Rafidah Mazlan, Sagshafraa Othman

**Affiliations:** 1 Faculty of Health Sciences, Audiology Programme, Center for Rehabilitation & Special Needs Studies, Universiti Kebangsaan Malaysia, Kuala Lumpur, Malaysia; 2 Faculty of Health Sciences, Center for Ear, Hearing and Speech, Universiti Kebangsaan Malaysia, Kuala Lumpur, Malaysia; UFPE: Universidade Federal de Pernambuco, BRAZIL

## Abstract

**Background:**

Childhood hearing loss is a significant health concern. Early identification and intervention are essential to maximize hearing potential and developmental outcomes, with primary care physicians (PCPs) playing a pivotal role in this process.

**Objectives:**

This study aimed to assess PCPs’ knowledge and attitudes toward childhood hearing loss, investigate the association between knowledge and attitudes, and examine the influence of demographic factors on PCPs’ knowledge and attitudes towards childhood hearing loss.

**Methods:**

This cross-sectional study was conducted from 30 November 2017 to 30 July 2018 at three public health clinics in Malaysia, specifically in the Federal Territory of Kuala Lumpur, Selangor, and Terengganu. A self-administered questionnaire assessed PCPs’ knowledge of general facts, diagnosis and intervention, and risk factors for childhood hearing loss. Additionally, the questionnaire evaluated PCPS’ attitudes across cognitive, affective, and behavioural domains regarding childhood hearing loss.

**Results:**

Most participants lacked sufficient knowledge about childhood hearing loss, with 61.4% not seeing it as a major health issue. Almost half (45.9%) didn’t know that children with hearing loss can succeed in regular schools, and 78% were unaware that hearing aids don’t fully restore normal hearing. Participants’ awareness of risk factors varied widely, ranging from 24.6% to 90.3%. Despite these knowledge gaps, participants generally had positive attitudes towards childhood hearing loss, especially in cognitive and behavioural aspects. The study found a strong positive link between knowledge and attitudes, but demographic factors didn’t significantly affect them.

**Conclusions:**

This study highlights the urgent need to address knowledge gaps among Malaysian PCPs regarding childhood hearing loss. While these knowledge gaps exist, PCPs’ positive attitudes form a foundation for developing targeted educational interventions to improve PCPs’ knowledge and skills in managing childhood hearing loss. Collaborative efforts are essential to translate these findings into meaningful improvements in paediatric audiological care.

## Introduction

Childhood hearing loss is a pressing global health concern affecting approximately 34 million children worldwide [1]. This condition, if left unaddressed, can profoundly impact children’s cognitive, linguistic, and socioemotional development [2,3]. Early hearing identification and intervention (EHDI) programs, including universal newborn hearing screening (UNHS), aim to detect hearing loss in infants by three months of age and initiate intervention by six months [4]. However, UNHS is not widely accessible in Malaysia [5,6]. Therefore, in developing countries like Malaysia, primary care physicians (PCPs) play a crucial role in identifying and intervening for childhood hearing loss as a first point of contact during well-child visits [7].

To effectively fulfil these roles, PCPs must have a comprehensive understanding and a favourable attitude toward childhood hearing loss. However, limited knowledge and unfavourable attitudes have been associated with delayed diagnosis and intervention, negatively impacting outcomes [8–14]. For example, only 58% were aware of hearing loss risks for infants in neonatal intensive care [9]. Additionally, only 11.5% indicated intention to refer children with confirmed hearing loss to an audiologist [14]. Investigations into PCP attitudes yield mixed results, with some studies showing positive perspectives but others with less favourable attitudes [9,11,12].

Currently, minimal research exists on Malaysian PCPs’ knowledge and attitude towards childhood hearing loss, which are vital to guide improvements in this setting. Therefore, the current study aimed to achieve three objectives: 1) To determine the level of knowledge and attitude of primary care doctors towards childhood hearing loss, 2) to investigate the association between knowledge and attitude towards childhood hearing loss among primary care doctors, and 3) to identify demographic variables associated with the knowledge and attitudes towards childhood hearing loss among primary care doctors in Malaysia.

The findings of this study underscore the pressing need for targeted educational and training initiatives to bolster the involvement of PCPs in managing childhood hearing loss in Malaysia. Additionally, the findings could inform strategies to enhance the identification and intervention processes for childhood hearing loss. This research represents a vital step towards improving paediatric audiological care in Malaysia.

## Methods and materials

This study received ethical approval the Research Ethics Committee of Universiti Kebangsaan Malaysia (UKM PPI/111/8/JEP 2017–709) and the Medical Research and Ethics Committee of the Ministry of Health Malaysia (NMRR-17-2965-38279). The research was conducted in accordance with the principles of the World Medical Association’s Declaration of Helsinki.

The cross-sectional study was carried out from 30 November 2017 to 30 July 2018 across three public health clinics located in Kuala Lumpur, Selangor, and Terengganu. These three states in Malaysia were chosen for their geographical representation of the major regions of Peninsular Malaysia. Kuala Lumpur and Selangor represent the West Coast, while Terengganu represents an East Coast state. The selection of these locations was also influenced by considerations of convenience.

Participants were PCPs employed full-time at the selected clinics, with a minimum basic medical degree. Prior to their participation, the PCPs received information about the study and provided informed consent. After consenting, participants completed a three-section survey. The signed consent form and the questionnaire were returned via postal mail.

The survey instrument was adapted from tools used in prior relevant literature [9,11,15]. To confirm the validity and reliability of the adapted tool, a pilot study was conducted with 17 PCPs at one clinic. Following constructive feedback, a questionnaire item was revised for clarity. The original statement, "According to recommended practice, children with congenital hearing loss should be diagnosed by three months old," was changed to "Diagnosis of hearing loss can be determined at three months old." The final adapted survey tool demonstrated high internal consistency with a Cronbach’s alpha of 0.8.

The questionnaire contained three sections. The demographic section collected data on age, gender, qualifications, and years in practice. The knowledge section had 22 multiple-choice items assessing comprehension of general facts, diagnosis/intervention guidelines, and risk factors for hearing loss in infants and toddlers from birth to 2 years old based on the Joint Committee on Infant Hearing recommendations. Responses in the knowledge section were scored dichotomously as either "Yes" (1 point) or "No" (0 points), with total possible scores ranging from 0 to 22. The attitude section used a 5-point Likert scale ranging from "0 = very likely" to "4 = not at all" for 25 statements, with possible scores ranging from 0 (minimum) to 100 (maximum). The final section of the questionnaire was composed of 25 items, each rated on a five-point Likert scale ranging from “0 = very likely” to “4 = not at all”. These items were designed to measure three domains of attitude: cognition (participants’ perceptions towards children with hearing loss), affective (participants’ feelings towards hearing-impaired children), and behavioural (participants’ actions or behaviours towards children with hearing loss). The total score for all items could range from 0 (minimum) to 100 (maximum).

### Data analysis

Descriptive analysis was performed using SPSS version 23.0, reporting mean, frequency, and percentage values. Negatively phrased items in the knowledge and attitude sections were reverse-coded to ensure that higher scores consistently reflect greater knowledge and more positive attitudes. A cut-off score of ≥80% correct responses was used to distinguish adequate from inadequate knowledge [16]. Mean Likert scale scores were calculated for overall attitude and subdomains. Scores below two indicated negative attitudes, while scores of two or more denoted positive attitudes. Chi-square tests assessed associations between categorical variables. Pearson’s correlation determined the relationship between knowledge and attitudes towards childhood hearing loss. Binary logistic regression analysis was used to examine the associations between knowledge, attitudes, and demographic factors, including gender, age, years in practice, and position. The a priori alpha level for statistical significance was set at 0.05.

## Results

### Participants

Out of 955 mailed questionnaires, 414 were returned, resulting in a response rate of 43.4%. The majority of participants were medical officers (92.5%), Malay (67.6%), and females (79.7%). The study primarily included young adults (88.2%). On average, participants had been practicing for 7.7 ± 5.9 years, with 77.5% having less than ten years of experience. experience or less. Further demographic details are presented in Table 1.

**Table 1:** Demographics of 414 participants.

Characteristic	Percentage (n = 414)
Gender	
Male	20.3 (84)
Female	79.7 (330)
Age Group	
20–40 years old	90.3 (374)
41–60 years old	9.7 (40)
Race	
Malay	67.6 (280)
Chinese	10.9 (45)
Indian	20.8 (81)
Other	0.7 (8)
Years in Practice	
1–10 years	77.5 (321)
>10 years	22.5 (93)
Qualifications	
Medical officer	92.5 (383)
Specialist (i.e., family medicine & public health)	7.5 (31)

### Overview of knowledge and attitude

Tables 2 and 3 present a detailed breakdown of the distribution of knowledge and attitudes across their respective domains. Most of the participants demonstrated inadequate knowledge regarding childhood hearing loss. The domain ’General Facts,’ as depicted in Table 2, revealed the highest percentage of participants who possessed adequate knowledge. Conversely, insufficient knowledge was prevalent across the remaining domains, with the risk factor domain displaying the most prominent deficiency.

**Table 2:** Knowledge distribution across domain and overall level.

Level of knowledge	Overall Knowledge (%, n)	General facts (%, n)	Diagnosis and intervention (%, n)	Risk factors (%, n)
Adequate (≥80%)	24.6 (102)	61.4 (254)	37.1 (153)	22.7 (94)
Inadequate (<80%)	75.4 (312)	38.8 (160)	62.9 (259)	77.3 (320)

**Table 3:** Mean Scores and percentage distribution across attitudes domain.

Domain	Mean (SD)	Attitude (%, n)	
		Positive	Negative
Affective (n = 413)	2.0 (0.8)	48.9 (202)	51.1 (211)
Cognitive (n = 407)	2.5 (0.9)	65.4 (266)	34.6 (141)
Behaviour (n = 407)	2.5 (1.2)	62.4 (254)	37.6 (153)

In the domain of attitudes, the participants generally exhibited positive attitudes towards childhood hearing loss, as reflected in an average attitude score of 2.3 ± 0.8. Moreover, a majority of participants (>60%) demonstrated favourable attitudes within the cognitive and behavioural domains, as detailed in Table 3.

Table 4 presents the distribution of correct responses across various questionnaire domains. Within the "General Facts" domain, there was a unanimous consensus regarding the pivotal role of hearing in children’s speech and language development (100%). Furthermore, an overwhelming majority underscored the significance of taking parental concerns about hearing loss seriously (99.5%). However, only 61.4% of participants agreed on the importance of childhood hearing loss as a health concern.

**Table 4:** Percentage and number of participants with correct answers for each question.

	Correct Answers	
**Knowledge items**	**n**	**%**
General facts on childhood hearing loss		
1. Hearing is important for children’s speech and language development.	414	100
2. Hearing loss in children is not an important health problem.	160	61.4
3. Parental suspicion of hearing loss should be taken seriously.	412	99.5
**Diagnosis and intervention**		
4. Diagnosis of hearing loss can be made at 3 months old.	338	81.6
5. Hearing aids are not suitable for babies less than one-year-old.	186	44.9
6. Hearing loss in children can only be diagnosed after age two.	162	39.1
7. Children with congenital hearing loss should start intervention by 6 months old.	349	84.3
8. All children with hearing loss need to attend special classes for the deaf.	224	54.1
9. Hearing aids restore hearing to normal in the way eyeglasses correct vision.	91	22.0
10. Fitting hearing aids in children with hearing loss is worth what it costs	395	95.4
**Risk factors for permanent hearing loss**		
11. *Meningitis*	374	90.3
12. Frequent cold	273	65.9
13. *Severe neonatal jaundice*	366	88.4
14. *Down syndrome*	261	63.0
15. *Cleft palate*	313	75.6
16. *>5 days in NICU*	102	24.6
17. *Birth weight <1*.*5kg*	286	69.1
18. Congenital heart disease	158	38.2
19. *Congenital rubella*	333	80.4
20. *Family history of childhood hearing loss*	347	83.8
21. *Congenital syphilis*	292	70.5
22. *Treated using aminoglycoside*	301	72.7

*Correct risk factors are indicated in italic.

In the "Diagnosis and Intervention" domain, the majority of participants (>80%) exhibited accuracy in responding to questions related to the optimal timeline for diagnosis and intervention (items 4 and 7). However, a decrease in consistency was noted when these items were presented as negative statements, resulting in slightly over 50% of participants providing accurate answers. Additionally, there appeared to be a lack of knowledge among participants concerning the potential for children with hearing loss to thrive in mainstream educational settings. Item nine had the lowest rate of correct responses, with only 22% of participants acknowledging that hearing aids do not fully restore normal hearing.

Within the risk factors domain, over 80% of participants correctly identified meningitis, severe neonatal jaundice, congenital rubella, and family history as factors associated with permanent hearing loss. However, other risk factors ranged from 25% to 73% correct, with the lowest score for neonatal stays over five days. In addition, a subset of participants demonstrated a misperception by erroneously associating frequent colds (34.1%) and congenital heart diseases (61.8%) with childhood hearing loss.

### Association between knowledge and attitude

The Pearson’s correlation analysis revealed a statistically significant positive correlation between knowledge and attitudes, with a correlation coefficient of r = 0.391 (N = 413, p < 0.01).

### Association of knowledge and attitude with demographic factors

The findings from the logistic regression analysis indicated that none of the examined demographic variables, including age, gender, years of practice, and designation, emerged as predictive factors for knowledge and attitude towards childhood hearing loss (refer to Table 5).

**Table 5:** Logistic regression analysis of socio-demographic factors predicting knowledge and attitude.

	Knowledge		Attitude	
Factors	Unstandardized Coefficient, B	*p*-value	Unstandardized Coefficient, B	*p*-value
Age	0.122	0.810	-0.057	0.911
Gender	0.478	0.150	0.480	0.055
Profession	-0.741	0.075	0.172	0.678
Years of Practice	0.571	0164	0.354	0.391

## Discussion

The present study represents a pioneering effort in examining the knowledge and attitudes of Malaysian PCPs regarding childhood hearing loss. Overall, the study’s results reveal that most participants had inadequate knowledge of childhood hearing loss. This knowledge deficit not only reflects a local concern but also resonates with global observations. Previous research conducted in diverse settings, including Nigeria [8], Mexico [11], and Jordan [14], has similarly underscored the prevalence of knowledge gaps in the domain of childhood hearing loss. These findings emphasize the global nature of this challenge, warranting comprehensive efforts for its resolution. The utilization of World Health Organization’s (WHO) training manuals on primary ear and hearing care [17] can serve as valuable resources for addressing these knowledge gaps through education. As recommended by the WHO, the urgency of developing educational programs and interventions to bridge these knowledge gaps and enhance the quality of care for children with hearing loss is paramount, regardless of their geographical location.

While knowledge deficits are acknowledged, a positive aspect emerges within the "General Facts" domain, where most participants exhibited adequate knowledge. This outcome indicates that PCPs generally have a good understanding of basic concepts in this domain. For instance, nearly unanimous recognition among PCPs of the significance of addressing parental concerns about their child’s hearing corresponds with prior observations, underscoring the important role of this aspect in driving referrals for audiological evaluations [11,12,18]. However, the fact that only 61.4% of participants recognized the importance of childhood hearing loss as a health concern indicates that there might be varying perceptions among the participants regarding the broader health implications of childhood hearing loss. These differing levels of agreement may stem from variations in PCPs’ understanding of the extent of the issue, including its prevalence, costs and cost/benefit ratio, inconsistent care co-ordination and system breakdowns, as reported by Moller et al. [19]. Further investigation into factors influencing this perception is warranted, as it could offer valuable insights for the development of targeted training programs tailored specifically to Malaysian PCPs.

In the domain of diagnosis and intervention, the study’s findings reveal both positive and concerning aspects. The high accuracy rate (>80%) in participants’ responses pertaining to the optimal timeline for diagnosis and intervention signifies PCPs’ awareness of the critical significance of early intervention, aligning well with the established best practices advocated by the Joint Committee on Infant Hearing [4]. However, the decreased in accuracy when items were presented as negative statements (e.g., hearing loss can only be diagnosed after two years old) suggests that participants may need help understanding these concepts. To address this gap, continuous education is necessary to enhance PCPs’ understanding and competence in handling these crucial concepts.

Furthermore, the lack of knowledge regarding the potential for children with hearing loss to thrive in mainstream educational settings echoes findings from previous research [13]. This finding highlights the need for interventions to encompass not only medical aspects but also broader considerations like educational inclusion and the capabilities of children with hearing loss. Additionally, only a minority of participants (22%) correctly understood the capabilities and limitations of hearing aids, which is consistent with the findings of Mazlan and Min’s study [20] involving healthcare professionals who directly manage children with hearing loss. This finding underscores the challenge non-audiology professionals may encounter in comprehending hearing aid concepts, emphasizing the importance of clearly communicating key points, including the inability of hearing aids to restore normal hearing or repair damaged hair cells.

The "Risk Factors" domain found that more than 80% correctly identified well-established risk factors associated with permanent hearing loss such as meningitis, severe neonatal jaundice, congenital rubella, and family history. Consistent findings were noted in other studies [9,10,12], further reinforcing the core understanding of these significant risk factors. However, participants’ responses concerning other risk factors, such as birth weight less than 1.5kg, cleft palate, and extended NICU stays exceeding 48 hours, displayed varying levels of understanding. This finding has important implications for early intervention efforts, as these risk factors could lead to hearing loss later in life [4]. Recognizing these factors becomes crucial for PCPs due to their pivotal role in continuous child development care, enabling them to effectively implement timely and targeted early intervention strategies and ensure optimal hearing health outcomes for children. Another important finding of this study is that some participants incorrectly associated factors such as frequent colds and congenital heart diseases with childhood hearing loss. Such misinformation is not unexpected, given similar knowledge gaps have been reported in previous studies involving healthcare professionals from various disciplines [9,21,22].

Despite knowledge deficits, the participants in this study demonstrated predominantly positive attitudes towards childhood hearing loss. This finding resonates with prior studies by Moller et al. [9], Yerraguntla et al. [12], and Kaspar et al. [23], highlighting the commitment exhibited by PCPs in the study to provide the best care and services to children with hearing loss.

The assessment of participants’ attitudes in this study, provides a comprehensive perspective, encompassing emotional, cognitive, and behavioural aspects, as advocated by Olson and Zanna [24]. The positive attitudes exhibited by a substantial proportion of participants, particularly in the cognitive and behaviour domains, indicate a general inclination towards empathy, understanding, and a readiness to engage with children who have hearing loss. These positive cognitive attitudes signify that PCPs possess a foundational knowledge of the importance of early identification, intervention, and comprehensive care for children with hearing loss. Moreover, the positive behavioural attitudes underscore PCPs’ willingness to translate their knowledge and emotional empathy into concrete actions that benefit children with hearing loss. It’s worth noting a slightly lower percentage within the affective domain. However, this percentage still falls within the positive range, indicating the potential for further development in cultivating emotional empathy and compassion toward children with hearing loss. Strategies such as incorporating experiential learning, role-playing, or exposure to patient narratives could be considered to enhance affective attitudes [25].

The statistically significant positive correlation between participants’ knowledge and attitudes towards childhood hearing loss suggests that as knowledge increases, attitudes become more positive. The positive correlation aligns with the theoretical underpinnings of attitudes, suggesting that individuals with a deeper understanding of a subject are more likely to develop positive attitudes towards it [26]. This outcome underscores the pivotal role of knowledge in shaping attitudes, particularly in the context of childhood hearing loss.

Despite the positive correlation between knowledge and attitudes towards childhood hearing loss, the examined demographic factors, such as age, gender, years of practice, and designation did not significantly influence knowledge and attitudes toward childhood hearing loss and aligns with findings from prior research. For example, Olusanya and Roberts (2006) found that knowledge gaps regarding infant hearing loss were evident across various demographics, suggesting that demographic factors may not always be strong predictors of knowledge deficits. Similarly, Moller et al. (2006) and Yerranguntla et al. (2016) observed that healthcare professionals across different demographics demonstrated a willingness to refer children for evaluation, indicating that attitudes may not be substantially influenced by demographic factors. These findings suggest that targeted interventions should be universally applied, rather than solely tailored to specific demographic groups.

The strength of this study lies in its pioneering investigation into the knowledge and attitudes of PCPs in Malaysia concerning childhood hearing loss, filling a significant research gap within this specific context. The comprehensive assessment of participants’ attitudes across emotional, cognitive, and behavioural domains enriches the understanding of PCPs’ perceptions towards childhood hearing loss. However, it is essential to acknowledge certain limitations in the study design. This study only included PCPs from three primary care clinics, which may restrict the generalizability of the findings. The cross-sectional design of the study captures attitudes and knowledge at a specific point in time, which limits the ability to observe changes or trends over an extended period. Additionally, the study’s reliance on self-reported data from participants introduces the potential for response bias, which may lead to an overestimation of positive attitudes due to participants’ desire to provide socially desirable responses.

To address these limitations, future studies could benefit from expanding the research to encompass larger, population-based groups of Primary Care Physicians (PCPs). This would improve the generalizability of the study’s findings. Such an expansion would likely yield more accurate and representative findings, thereby enhancing the robustness of the study’s conclusions. Additionally, adopting longitudinal designs to track changes in PCPs’ knowledge and attitudes over time, particularly in response to targeted educational interventions, would offer valuable insights into the effectiveness of such interventions.

## Conclusions

This study identified gaps in knowledge and positive attitudes about childhood hearing loss among Malaysian PCPs. These global knowledge deficits urgently necessitate comprehensive training programs. However, the physicians’ receptiveness suggests interventions can enhance early identification and care for affected children. Relevant stakeholders must act on these findings to advance paediatric audiological care.

## Supporting information

S1 Data Minimal data set: This file contains the raw data collected during our study, specifically the responses from participants regarding their knowledge and attitudes on the subject matter of our research. (XLSX)
